# Mirodenafil improves cognitive function by reducing microglial activation and blood–brain barrier permeability in ApoE4 KI mice

**DOI:** 10.3389/fnagi.2025.1579411

**Published:** 2025-05-15

**Authors:** Yejin Park, Subin Moon, Harry Jung, Songyi Park, Ju Won Kim, Dan-Gyeong Song, Yong-Ho In, Sang Won Han, Jong-Hee Sohn, Chan Hee Lee

**Affiliations:** ^1^Department of Biomedical Science, Hallym University, Chuncheon, Republic of Korea; ^2^Institute of New Frontier Research Team, College of Medicine, Hallym University, Chuncheon, Republic of Korea; ^3^AriBio Co., Ltd., Seongnam-si, Gyeonggi-Do, Republic of Korea; ^4^Department of Neurology, Chuncheon Sacred Heart Hospital, Hallym University College of Medicine, Chuncheon, Republic of Korea; ^5^Program of Material Science for Medicine and Pharmaceutics, Hallym University, Chuncheon, Republic of Korea

**Keywords:** Alzheimer’s disease, human Apolipoprotein E, cerebrovascular perfusion, cognitive function, mirodenafil

## Abstract

**Introduction:**

Alzheimer’s disease (AD) has significant public health concerns in the aging society. AD can compromise brain function and lead to severe neurological abnormalities associated with dementia. The human Apolipoprotein E (ApoE4) gene is a strong risk factor for AD. However, comprehensive analyses and improvements of mouse models expressing ApoE4 remain largely unexplored.

**Methods:**

ApoE4 knock-in (KI) mice were used to investigate the role of humanized ApoE4 in hippocampal histological changes and cognitive impairment. Cerebrovascular perfusion, blood–brain barrier (BBB) integrity, microgliosis, and amyloid-beta 42 (Aβ_42_) accumulation were examined. Cognitive functions were assessed using the Morris water maze, Y-maze, and novel object recognition tests. Mirodenafil, a potent and selective phosphodiesterase 5 inhibitor (PDE5i), was orally administered to ApoE4 KI mice for 4 weeks. An *in vitro* BBB model and BV2 microglial cells were used to investigate endothelial permeability and inflammation.

**Results:**

ApoE4 KI mice exhibited not only reduced cerebrovascular perfusion and CLN-5 expression but also increased microgliosis and Aβ_42_ accumulation in the hippocampus. These phenomena were accompanied by impaired cognitive functions. Mirodenafil administration reversed the histological and behavioral alterations induced by ApoE4 KI. *In vitro*, mirodenafil treatment mitigated Aβ_42_-induced endothelial permeability and lipopolysaccharide-induced microglial inflammation.

**Discussion:**

These findings suggest that mirodenafil enhances cerebrovascular function, preserves BBB integrity, and mitigates neuroinflammation in ApoE4 KI mice, leading to cognitive improvement. PDE5 inhibition may serve as a promising therapeutic approach for addressing ApoE4-associated cerebrovascular and cognitive dysfunction.

## 1 Introduction

Alzheimer’s disease (AD) is a prevalent neurodegenerative disease characterized by cognitive decline, memory loss, changes in mood and personality, and difficulty with communication (Kumar et al., 2024). These symptoms are mainly due to increased neuroinflammation and neuronal dysfunction; however, the specific causes and therapeutic strategies remain unknown despite extensive research efforts (Heneka et al., 2015). Between 2000 and 2019, deaths from AD increased by 145%, and it is estimated that the number of dementia patients in the United States will rise to 13.8 million by 2060 without significant medical breakthroughs (2023). Furthermore, the incidence and mortality rates of dementia, which increase with age, are expected to continue rising sharply in line with the aging society.

Clinical studies and a meta-analysis of genome-wide association studies (GWAS) have demonstrated the strong association of apolipoprotein E (ApoE) with cognitive decline and the accumulation of amyloid-beta 42 (Aβ_42_) in patients with AD (Corder et al., 1993; Bellenguez et al., 2022; Im and Choi, 2024). In particular, the ε4 allele of the Apolipoprotein E (APOE4) gene stands out as the most significant genetic risk factor for late-onset AD; the ε2 allele has been identified as neuroprotective (Van Cauwenberghe et al., 2016; Corder et al., 1994; Corder et al., 1993). However, there is a difference between mouse and human APOE genes. Human ApoE has three isoforms, ApoE2, ApoE3, and ApoE4, whereas mice express only one type of ApoE (Rajavashisth et al., 1985). Among the isoforms in humans, ApoE4 is strongly associated with an increased risk of AD, whereas the other APOE isoforms or mouse ApoE do not directly induce AD or have lower AD risk (Troutwine et al., 2022). Therefore, researchers utilized a method wherein the human ApoE4 gene was knocked into the mouse ApoE gene, creating an ApoE4 KI mouse model (Leung et al., 2012; Tong et al., 2016). However, the specific mechanisms by which ApoE4 KI leads to the AD progression is not well understood.

Cerebral hypoperfusion plays a crucial role in cognitive decline and the progression of AD, a condition referred to as vascular cognitive impairment (VCI) (Rajeev et al., 2023). Vascular dysfunction is actively being investigated as a therapeutic target, as it represents a potentially modifiable factor before the onset of dementia (Wentzel et al., 2001). However, studies demonstrating the improvement of AD through cerebrovascular stabilization remain highly limited. A recent study showed that inhibiting vascular endothelial growth factor (VEGF) with bevacizumab can improve early-stage cerebrovascular dysfunction and enhance cognitive function (Zhang et al., 2024). Another recent study demonstrated that preventing blood clotting with tissue plasminogen activator (tPA) can mitigate cerebral amyloid angiopathy induced by Aβ and improve cognitive function (Uekawa et al., 2024). These findings suggest that vascular-stabilizing agents could be a promising strategy for treating AD.

Clinical reports have demonstrated a close association between the ApoE4 allele and the development of vascular dementia (Davidson et al., 2006; Luo et al., 2017). These findings suggest that vascular stabilization and improved blood flow may enhance cognitive function in ApoE4-associated AD. Based on this evidence, we investigated the role of phosphodiesterase 5 inhibitors (PDE5is), which are known to influence vascular dilation, maintenance, and stabilization, as a therapeutic option. Mirodenafil is a highly selective PDE5i, which has demonstrated significant improvement in erectile dysfunction (Park et al., 2014). Several PDE5 inhibitors, including sildenafil, vardenafil, and tadalafil, are recently being studied for their potential to improve AD (Hainsworth et al., 2023). Mirodenafil is currently in Phase 3 clinical trial for early AD (NCT05531526). Moreover, *in vivo* studies using NSE/APP-C105 transgenic mice, mirodenafil reduces Aβ_42_ burden and improves cognitive function (Kang et al., 2022). *In vitro* studies using SH-SY5Y neuroblastoma and HT-22 mouse hippocampal cell lines have shown that mirodenafil treatment inhibits Aβ_42_-induced cell death (Kang et al., 2022). These results strongly suggest that mirodenafil has promising potential as a future treatment modality for AD, although the mechanisms by which it could improve AD are not fully understood.

In the present study, we investigated (1) the histological and behavioral changes in ApoE4 KI mice compared to age-matched WT mice, (2) the effects of mirodenafil on cerebrovascular perfusion, inflammation, and cognitive function in ApoE4 KI mice, and (3) the underlying mechanisms.

## 2 Materials and methods

### 2.1 Cell culture

BV2 cells were cultured in Dulbecco’s Modified Eagle Medium (DMEM) medium (Cytiva, #SH30022.01) supplemented with 10% fetal bovine serum (FBS; Cytiva, #SV30207.02) and 1% penicillin/streptomycin (P/S; Gibco, #15140–122). Before drug treatment, the cells were plated in a 6-well plate at a density of 5 × 10^5^ cells per well. After 24 h, the cells were incubated in DMEM medium (1% FBS, 1% P/S) with lipopolysaccharide (100 ng/mL, Invitrogen, #00-4976-93) and/or mirodenafil for 24 h.

### 2.2 Mice

Apoe^tm1.1(APOE*4)Adiuj^ (ApoE4 knock-In [KI]; C57BL/6J background) male and female mice were purchased from Jackson Laboratory (#027894) and maintained at a specific pathogen-free (SPF) animal facility. These mice carry the human ApoE4 gene (148 bp) compared to the wild-type (WT) gene (224 bp) (Supplementary Figure 1). Seven-week-old male C57BL/6J mice were purchased from DBL (Chungbuk, Korea) for age-matched wild-type (WT) mice and maintained under the same housing conditions as ApoE4 KI mice. Mice were housed in a temperature-controlled room (22 ± 1°C) with a 12 h light–dark cycle (lights on at 8 a.m. and off at 8 p.m.). Mice were freely provided a chow diet (CD) (Cargil Agri Purina, #EEGJ30060) and water.

### 2.3 Drug administration and experimental design

Mirodenafil-2HCl was dissolved in distilled water (DW). A dosage of 6 mg/kg mirodenafil was orally administered to approximately 11–13 month-old mice daily for 4 weeks. The oral dose of 6 mg/kg mirodenafil in the mice is equivalent to the oral dose of 30 mg used in the clinical trial (Kang et al., 2020; Kang et al., 2023). The control group was provided with an equivalent amount of DW for the same period. After 4 weeks of mirodenafil administration, behavior tests and histological analysis were performed on ApoE4 KI and age-matched WT mice.

### 2.4 *In vivo* vascular perfusion test

To confirm vascular perfusion, 150 kD Fluorescein Isothiocyanate (FITC)-Dextran (Sigma, #46946-100MG) was purchased from Sigma. Mice received 10 mg of FITC-Dextran through the tail vein. After 3 min, the mouse brain was rapidly obtained from the mouse and stored in 4% paraformaldehyde for 1 day, followed by incubation in 30% sucrose solution for dehydration for 2 days. The brain samples were coronally sectioned at a 40 μm thickness using a cryostat (Leica, Wetzlar, Germany). One out of every eight slices were examined or stored at-80°C. FITC fluorescence on one of every eight slices was performed under confocal (Zeiss 710) or fluorescence (Zeiss Axioscope 5, #430035-9061-00) microscopy. Analysis was conducted using ZEN blue edition (Zeiss, Ver. 2.6) and Photoshop (Adobe Systems, Ver. 21.0.2).

### 2.5 *In vitro* vascular permeability test

The *in vitro* vascular permeability tests were conducted based on a previous study (Park et al., 2023). Sterile Transwell Polycarbonate Membrane Insert (12 wells, 0.4 μm pore size, SPL Biosciences, #37012) was used for this experiment. The b.End.3 endothelial cells were seeded at a density of 5 × 10^4^ cells/cm^2^ onto 12-well transwell semi-permeable supports. C8-D1A astrocyte cells were seeded at a density 2.5 × 10^4^ cells/cm^2^ onto lower chamber of the transwell. The b.End.3 cells were cultured in 10% FBS DMEM at 37°C in a 5% CO_2_ incubator. Once the cells reached confluence, they were incubated in 1% FBS DMEM and then treated with 10 mM Aβ_42_ and 5 mM or 10 mM mirodenafil for 12 h or 24 h. To examine endothelial cell permeability, FITC-dextran (30 mg/mL, Thermo Fisher, #J14495) was added to the upper chamber and incubated for 30 min. Absorbance was then measured at 492 nm (excitation) and 520 nm (emission) with the medium in the lower chamber using a FLUOstar Omega microplate reader. The transendothelial electrical resistance (TEER) was measured using a chopstick electrode (World Precision Instruments, #STX2) and a Millicell ERS-2 volt/*Ω* meter (Millipore). The results were expressed as Ω × cm^2^.

### 2.6 Immunostaining

Mice were perfused with 50 mL cold saline and 50 mL cold 4% paraformaldehyde under anesthesia using isoflurane (Hana Pharm Co., Ltd). Whole brains were obtained and fixed with 4% paraformaldehyde at 4°C for 16 h and dehydrated in PBS-based 30% sucrose solution until the brain sank. Brains were sectioned using a cryostat (Leica, Wetzlar, Germany). For Aβ_42_ staining, the brain tissues were incubated in 3% bovine serum albumin (BSA) in 0.3% PBS-triton X-100 (PBST) at room temperature (RT) for 1 h unless indicated otherwise. Subsequently, the slices were incubated with Aβ_42_ antibody (1:1,000, Abcam, #ab201060) at 4°C for 16 h. For hippocampal blood vessel staining, brain slices were incubated with primary antibodies against CD31 (1:200, BD Pharmingen, #550274), Claudin-5 (CLN-5; 1:500, Thermo Fisher, #34–1,600) at 4°C for 16 h and then at RT for 1 h. For hippocampal microglia staining, brain slices were incubated with primary antibodies against Iba1 (1:400, Abcam, #ab5076), inducible nitric oxide synthase (iNOS; 1:400, BD Biosciences, #610328), and Arginase-1 (Arg-1; 1:400, Abcam, #ab91279) at 4°C for 16 h and then at RT for 1 h. After washing, the brain slices were incubated with the appropriate Alexa-Fluor 488-, 555-conjugated secondary antibodies (1:1,000, Invitrogen, #A21206, #A2633526, #A2604365, and #A21428) at RT for 1 h. For nuclear staining, Brain slides were incubated with DAPI (1:10,000, Sigma, #D9542) for 10 min. After mounting with mounting solution (DAKO, #S3025), fluorescence images were taken by confocal or fluorescence microscopy. Quantification of fluorescence intensity and cell counting were performed using ImageJ (NIH, Ver. 1.8.0) and Photoshop.

### 2.7 Quantitative PCR analysis

For mRNA analysis, total RNA was extracted from BV2 cells using the easy-BLUETM Total RNA Extraction Kit (INTRON, #17061), and cDNA was synthesized. Quantitative Real-Time PCR was conducted using the SYBR green (Applied BiosystemsTM, #4367659). Primers used in this study (*Il-1β*, *Il-6*, *Tnfα*, *inos,* and *β-actin*) are provided in Supplementary Table 1. The quantitative analysis was examined by ^∆∆^CT method, and each mRNA expression level was normalized to *β-actin*.

### 2.8 Behavioral tests

For the Morris Water Maze (MWM) test, a 100 cm-diameter circular tank containing a platform with a diameter of 8 cm is filled with water (21 ~ 22°C) dissolved with non-toxic white paint 1 cm above the height of the platform. For training of the mice, mice are placed on the platform for 3 s to recognize the visual cues placed on the four walls of the tank. Subsequently, the mice were placed at a specific location apart from the platform. The time taken to reach the hidden platform, the distance traveled, and the swimming speed was measured and analyzed over 60 s. All movements were recorded using video-tracking software (Noldus EthoVision XT, Leesburg, VA, USA). The experiment was conducted over six consecutive days.

For Y-maze test, A Y-maze with three arms designated as A, B, and C was used. Dimensions of each arm were 38 × 15.5 × 4 cm (length × height × width). Mice were placed at the end of arm C and their movements were measured for 5 min. Using the tracking system, All arms and their entry points were video-tracked and automatically analyzed. The total number of times the mice entered each of the three arms was divided by the total number of entrances to calculate the ratio of entries per arm, which was named spontaneous alternation. The formula is as follows: Spontaneous alternation = (number of spontaneous alternations/total number of arm entries – 2) X 100.

For the novel object recognition (NOR) test, two identical objects (designated as A and A’) were placed in an open arena with dimensions of 50 cm in length, width, and height. The mice were placed and adapted for 10 min in the arena, which was conducted twice a day with a 4 h interval. The adaptation was conducted over six consecutive days. After replacing the first object (A’) with a different one designated as B, the cognitive function toward the novel object (B) was examined. This experiment was also recorded using video-tracking software.

### 2.9 Western blotting

Cells were lysed in radioimmunoprecipitation assay (RIPA) buffer (Biosesang, #RC2002-050-00) supplemented with protease inhibitors (GenDEPOT, #P3100) and phosphatase inhibitors (GenDEPOT, #P3200). The hippocampal tissues of WT and ApoE4 KI mice were collected 15 min after oral administration of mirodenafil. The tissues were lysed in the same manner as described above. The lysates were then centrifuged at 13,000 rpm for 30 min at 4°C. Protein samples were loaded onto a 12% SDS-PAGE gel and subsequently transferred to a polyvinylidene difluoride (PVDF) membrane (Millipore, #IPVH00010). The membranes were blocked for 1 h in 3% bovine serum albumin (Bovogen, #BSAS0.1) prepared in 1x TBST buffer (Tween 20, Tris, and NaCl). After blocking, the membranes were incubated overnight at 4°C with primary antibodies targeting CLN-5 (1:1000, Invitrogen, #34–1600), pCREB (1:1000, Cell Signaling, #9198S), tCREB (1:1000, Cell Signaling, #9197S), BDNF (1:1000, Invitrogen, #PA5-85730) and β-actin (1:1000, Cell Signaling, #3700S). Following washes with 1x TBST buffer, the membranes were incubated for 1 h at RT with HRP-conjugated secondary antibodies, including anti-rabbit IgG (1:1000, Cell Signaling, #7074) and anti-mouse IgG (1:1000, Cell Signaling, #7076). A chemiluminescence imaging system (GE Healthcare, ImageQuant LAS 500) was used for protein detection. Band quantification was performed using ImageJ software (Ver. 1.8.0).

### 2.10 Statistical analysis

All data are presented as mean ± standard error of the mean (SEM). Statistical analyses were performed using Prism software (GraphPad, Ver. 10.2.2). Statistical significance among the groups was tested using one-way or two-way analysis of variance (ANOVA) followed by a post-hoc least significant difference (LSD) test or a two-sided Student’s *t*-test or simple linear regression, if appropriate. Statistical significance was defined at *p* < 0.05.

## 3 Results

### 3.1 Reduced cerebrovascular perfusion was observed in ApoE4 KI mice

**Figure 1:**
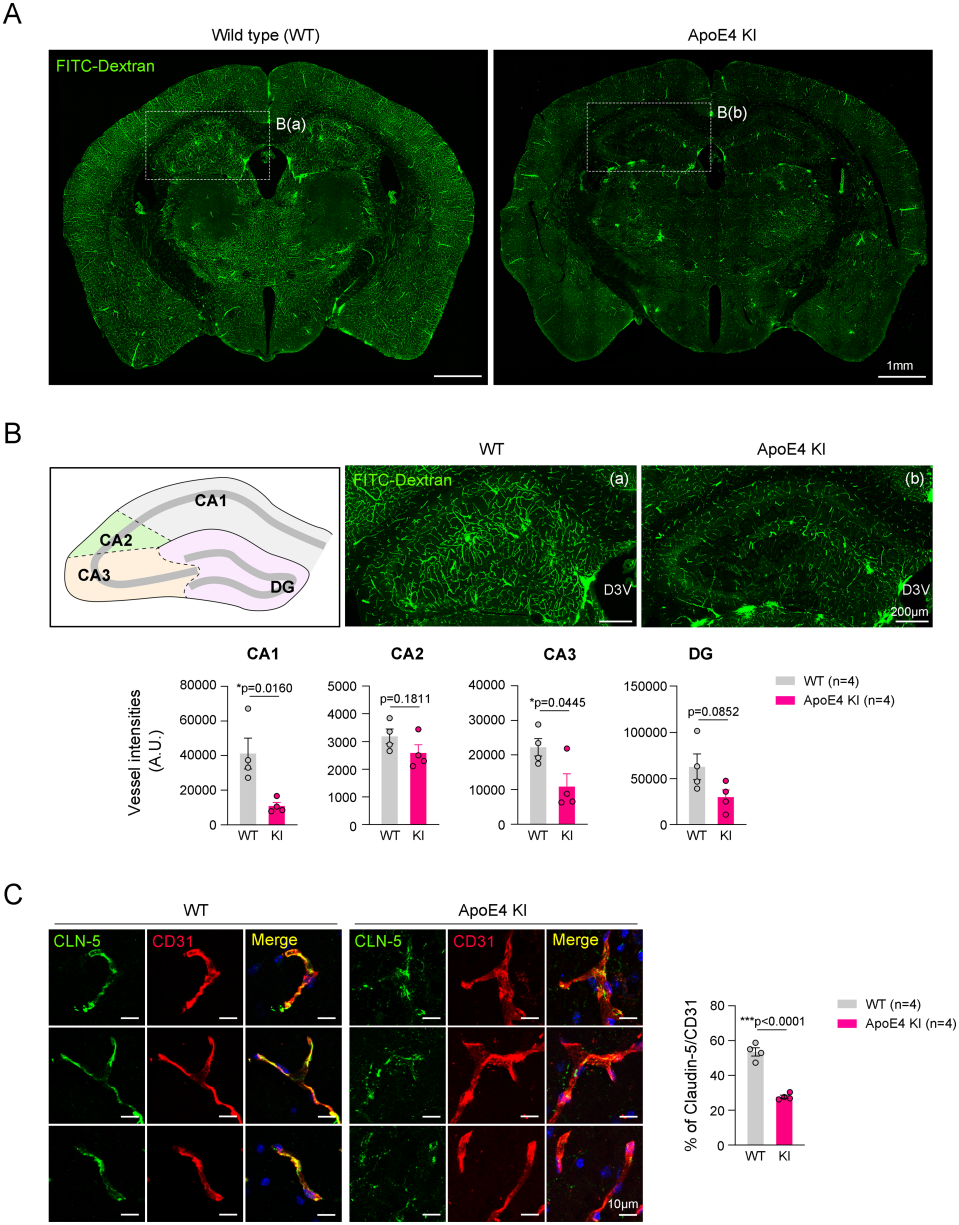
Reduced vascular perfusion and integrity in the hippocampus of the ApoE4 KI mouse. **(A)** Representative images showing vessel intensities using FITC-Dextran in WT and ApoE4 KI mice. Scale bars, 1 mm. **(B)** Representative images and measurement graphs showing the distribution and intensity of FITC-Dextran+ vessels in the hippocampus of WT and ApoE4 KI mice (*n* = 4). Scale bars, 200 μm. **(C)** Representative images and measurement graphs of double immunostaining images for CD31 and CLN-5 in the hippocampus of WT and ApoE4 KI mice (*n* = 4). Scale bars, 10 μm. Results are presented as mean ± SEM. Results are presented as mean ± SEM. Statistics were performed using two-sided Student’s *t*-test **(B)**. *p < 0.05, ***p < 0.001 between the indicated groups.

Cerebral hypoperfusion and impaired blood–brain barrier (BBB) integrity are associated with AD pathogenesis (Di Marco et al., 2015). A previous study showed that FITC-conjugated dextran (FITC-dextran) was used to determine the reduced cerebral perfusion and increased non-perfused lesion by angiopathy (Tan et al., 2015; Ida et al., 2018). To compare the cerebrovascular perfusion between ApoE4 KI and age-matched WT mice, we intravenously injected FITC-Dextran into these mice. Fluorescence images showed that the intensities of the FITC-Dextran tracer were significantly decreased throughout the brain of ApoE4 KI male mice compared with WT mice (Figure 1A). This phenomenon was similarly observed in male and female ApoE4 KI mice (Supplementary Figure 2). Notably, in the hippocampus, vascular perfusion in the cornu ammonis 1 (CA1) and CA3 regions significantly decreased in ApoE4 KI male mice compared with WT mice, whereas the CA2 and dentate gyrus (DG) regions showed a decreasing trend in ApoE4 KI male mice (Figure 1B). To investigate the reason for reduced cerebrovascular perfusion in the hippocampus of ApoE4 KI male mice, we examined the intensity of claudin-5 (CLN-5), a tight junction (TJ)-associated protein in the blood vessels. Our immunostaining data showed a decrease in the intensity of CLN-5 in the hippocampal blood vessels of ApoE4 KI male mice compared with that of WT mice (Figure 1C). These findings indicated that humanized ApoE4 KI impairs cerebrovascular perfusion in the hippocampus by lowering vascular integrity.

### 3.2 Increased microgliosis and Aβ_42_ accumulation were observed in the hippocampus of ApoE4 KI mice

**Figure 2:**
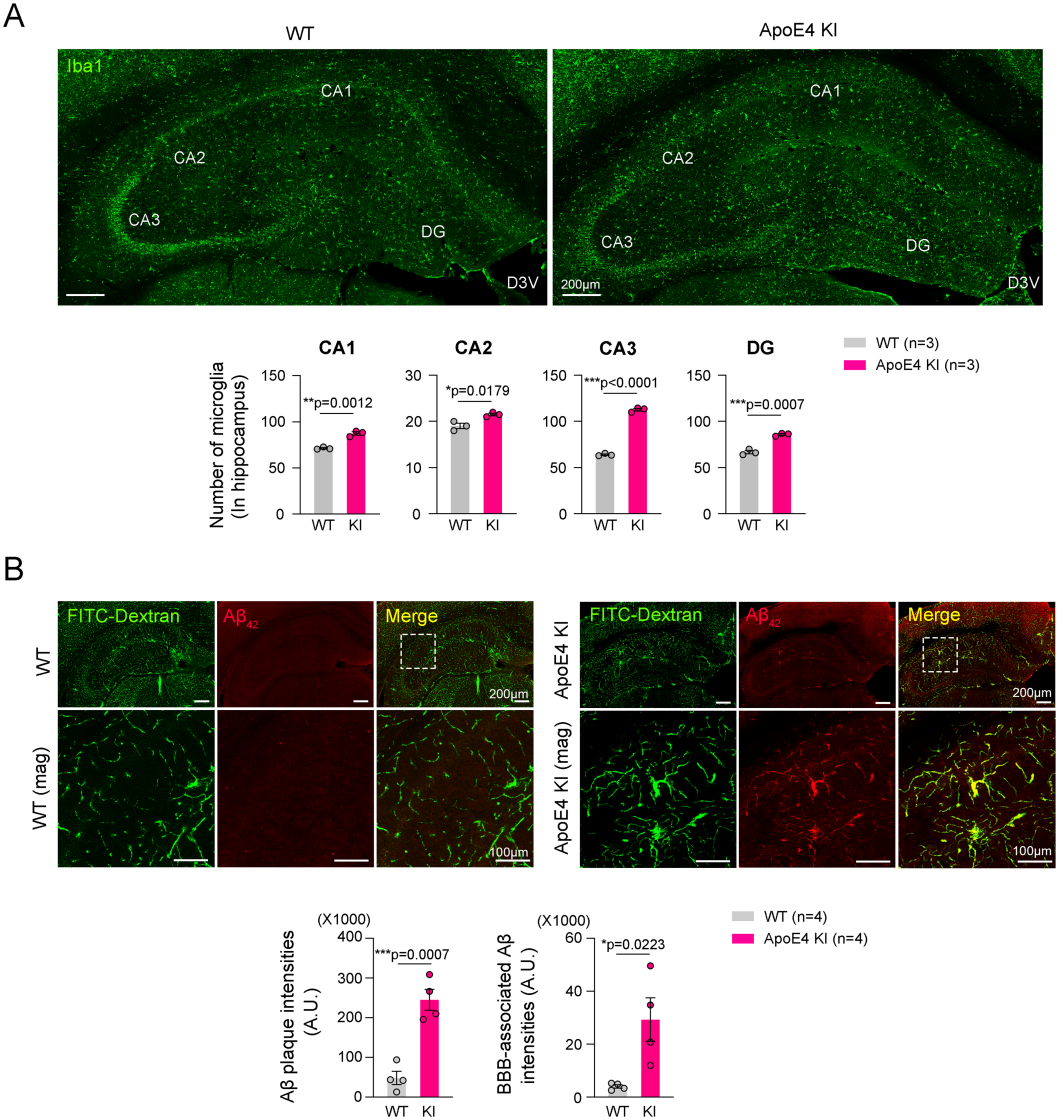
Reduced cerebrovascular stability in the hippocampus of ApoE4 KI mice. **(A)** Representative images and quantification of double immunofluorescence of Iba1 in the hippocampus of WT and ApoE4 KI mice (*n* = 3). Scale bars, 200 μm for images. **(B)** Representative images and quantification of double immunofluorescence of FITC-Dextran and Aβ_42_ in the hippocampus of WT and ApoE4 KI mice (*n* = 4). Scale bars are 200 μm for low-magnification images and 100 μm for high-magnification images. Statistics were performed using two-sided Student’s *t*-test **(A,B)**. *p < 0.05, **p < 0.01, and ***p < 0.001 between the indicated groups.

Microgliosis and Aβ_42_ accumulation in the hippocampus have been considered a hallmark of AD pathogenesis (Zhang Y. et al., 2023; Hansen et al., 2018). We examined hippocampal microgliosis in age-matched WT mice and ApoE4 KI mice. The results showed a significant increase in microgliosis throughout the hippocampus of ApoE4 KI mice compared to age-matched WT mice (Figure 2A). However, the correlation data showed that reduced cerebrovascular perfusion and microgliosis exhibited a similar pattern to the data observed in Figure 1B (Supplementary Figure 3). These results suggest that microgliosis may precede cerebrovascular hypoperfusion in the progression of AD in ApoE4 KI mice.

Recent study reported that Aβ_42_ accumulates around hippocampal blood vessels (Oddo et al., 2009). We next examined the Aβ_42_ accumulation and its distribution in the hippocampus. Consistently, our immunostaining data showed that Aβ_42_ was significantly increased in the hippocampus of ApoE4 KI mice and was barely seen in those of WT mice (Figure 2B). Moreover, the Aβ_42_ was predominantly observed in the hippocampal blood vessels (Figure 2B). These results suggested that humanized ApoE4 KI contributes to Aβ_42_ accumulation in the hippocampus.

### 3.3 Reduced cognitive function occurs in the hippocampus of ApoE4 KI mice

**Figure 3:**
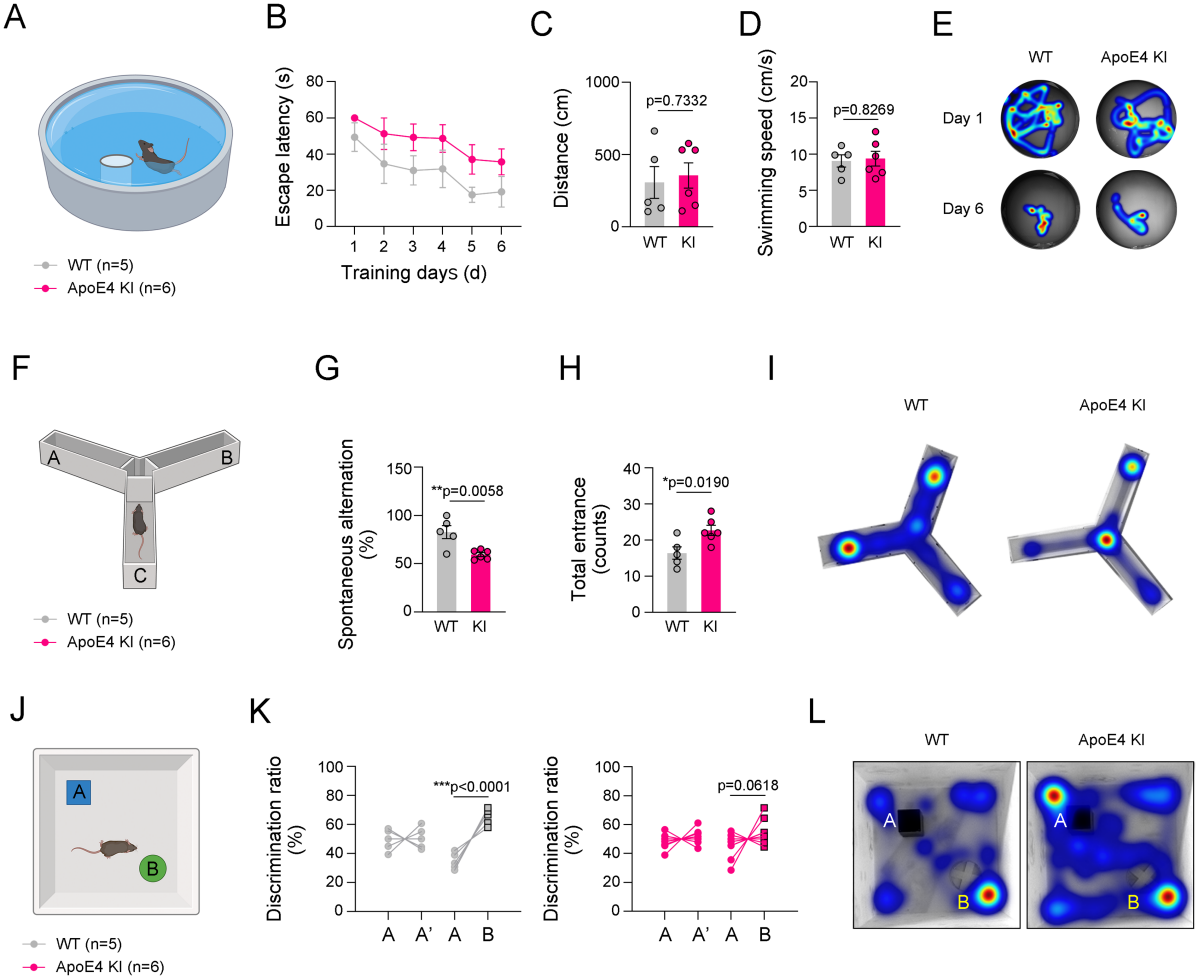
Impaired cognitive function in ApoE4 KI mice. **(A)** Illustration of Morris Water Maze (MWM). **(B–D)** The escape latencies, distances, and swimming speed over 6 consecutive training days in WT and ApoE4 KI groups (*n* = 5 for WT group and *n* = 7 for ApoE4 KI group). **(E)** Representative swimming paths between WT and ApoE4 KI groups at 1st day and 6th day from the first training. **(F)** Illustration of Y-maze. **(G,H)** Percentage of spontaneous alteration and total counts of entrance in WT and ApoE4 KI groups (*n* = 5 for WT group and *n* = 7 for ApoE4 KI group). **(I)** Representative movement paths between WT and ApoE4 KI groups. **(J)** Illustration of Novel Object Recognition (NOR) test. **(K)** Discrimination ratio (%) before and after replacing with a novel object in WT and ApoE4 KI groups (*n* = 5 for WT group and *n* = 7 for ApoE4 KI group). **(L)** Representative movement paths between WT and ApoE4 KI groups. Results are presented as mean ± SEM. Statistics were performed using two-sided Student’s *t*-test **(C,D,G,H,K)** and two-way ANOVA **(B)** followed by *post hoc* LSD test. *p < 0.05, **p < 0.01, and ***p < 0.001 between the indicated groups. The illustrations provided in panel **(A,F,J)** were created in BioRender.com.

AD-induced histological alterations, including reduced cerebrovascular perfusion, microgliosis, and Aβ accumulation, are closely associated with cognitive impairment (Barisano et al., 2022; Mattsson et al., 2014; Marchant et al., 2013). We investigated whether cognitive and memory performance was impaired in ApoE4 KI mice. To examine this, we performed MWM (Figure 3A), Y-maze (Figure 3F), and NOR tests (Figure 3J) in ApoE4 KI mice and age-matched WT mice. These three experiments are widely used to measure the comprehensive cognitive function or memory in mice, with MWM assessing spatial learning and memory (Vorhees and Williams, 2006), Y-maze evaluating spatial short-term memory (Kraeuter et al., 2019), and NOR assessing cognitive function related to novel objects (Grayson et al., 2015). In the MWM test, ApoE4 KI mice did not show significant changes in the time to find the platform or swimming speed compared to age-matched WT mice (Figures 3B–E). However, in the Y-maze test, a significant decrease in spontaneous alternation relative to total entrances was observed in ApoE4 KI mice (Figures 3G–I). Moreover, in the NOR test, ApoE4 KI mice showed decreased recognition of new objects compared to age-matched WT mice (Figures 3K,L). Their results indicate that cognitive function and short-term memory are impaired in humanized ApoE4 KI mice.

### 3.4 Mirodenafil ameliorated histological changes in the hippocampus of ApoE4 KI mice

**Figure 4:**
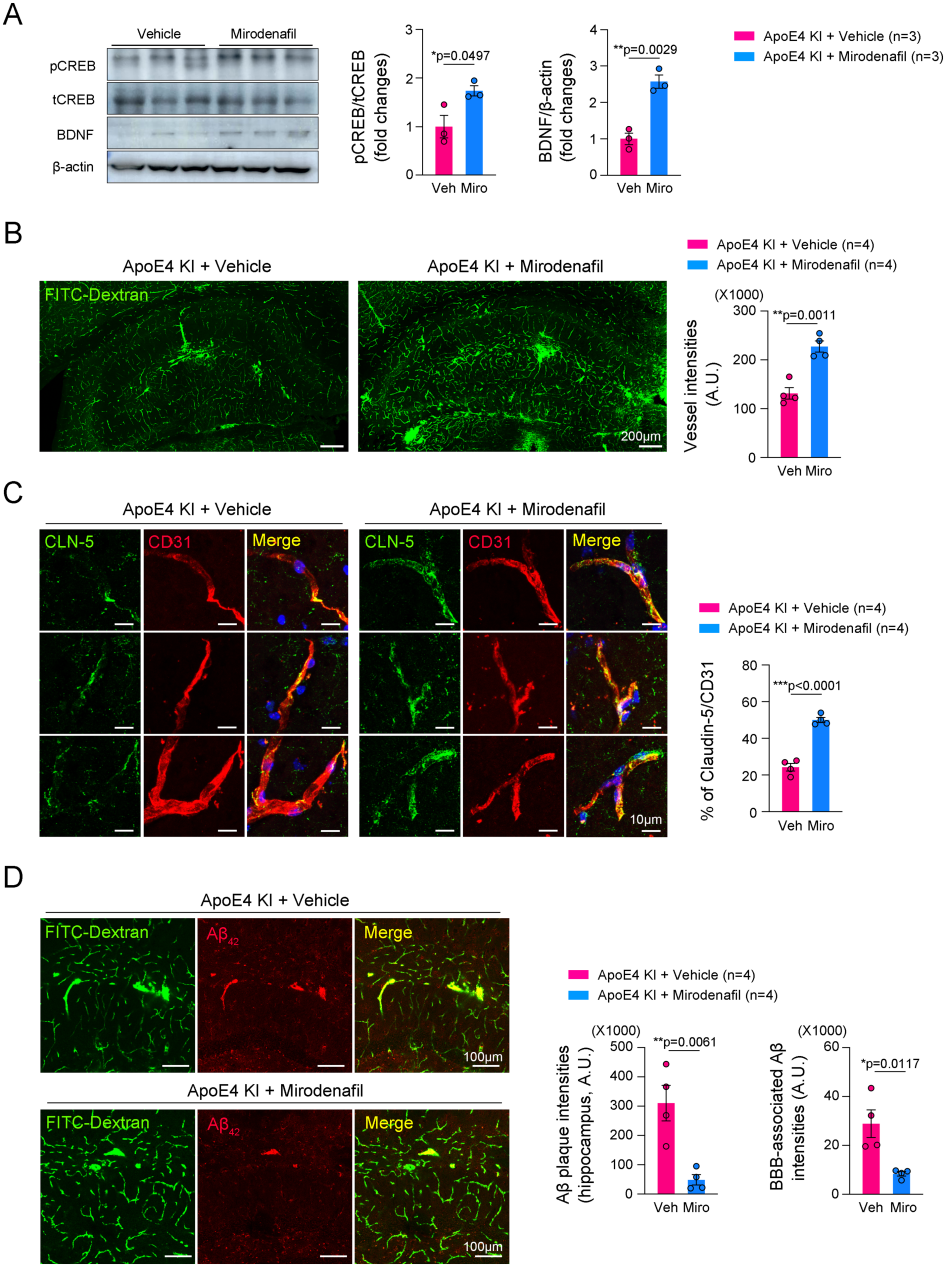
Mirodenafil improves cerebrovascular perfusion and reduces Aβ_42_ accumulation in the hippocampus of ApoE4 KI mice. **(A)** Western blotting data and quantification of pCREB, tCREB, and BDNF in the hippocampus of WT and ApoE4 KI mice (*n* = 3). **(B)** Representative images and quantification of double immunostaining for CD31 and CLN-5 in the hippocampus of vehicle- or mirodenafil-administered ApoE4 KI mice (*n* = 4). Scale bars, 200 μm. **(C)** Representative images and quantification of FITC-Dextran in the hippocampus of vehicle- or mirodenafil-administered ApoE4 KI mice (*n* = 4). Scale bars, 10 μm. **(D)** Representative images and quantification of Aβ_42_ in the hippocampus of vehicle- or mirodenafil-administered ApoE4 KI mice (*n* = 4). Scale bars, 100 μm. Results are presented as mean ± SEM. Statistics were performed using two-sided Student’s *t*-test **(A–D)**. *p < 0.05, **p < 0.01, and ***p < 0.001 between the indicated groups.

PDE5i has been known to not only enhance blood perfusion in cerebrovascular diseases such as ischemic stroke and subarachnoid hemorrhage (Ölmestig et al., 2020; Dhar et al., 2016), but also reduce the risk of AD (Hainsworth et al., 2023). We tested whether mirodenafil improves histopathological changes induced by humanized ApoE4 KI. Consistent with a previous report (Kang et al., 2022), mirodenafil administration increased CREB phosphorylation and brain-derived neurotrophic factor (BDNF) expression in the hippocampus of ApoE4 KI mice (Figure 4A). Mirodenafil administration for 4 weeks significantly improved cerebrovascular perfusion in the hippocampus of ApoE4 KI mice compared with vehicle-administered ApoE4 KI mice (Figure 4B). Moreover, mirodenafil enhanced the expression of CLN-5 in the hippocampal blood vessels (Figure 4C). We next investigated whether mirodenafil reduces Aβ_42_ accumulation in the hippocampus of ApoE4 KI mice. Our immunofluorescence data showed that mirodenafil suppressed Aβ_42_ accumulation around blood vessels (Figure 4D). These results indicated that mirodenafil improves cerebrovascular perfusion and attenuates vessel-associated Aβ_42_ accumulation in ApoE4 KI mice.

### 3.5 Mirodenafil improved cognitive function in ApoE4 KI mice

**Figure 5:**
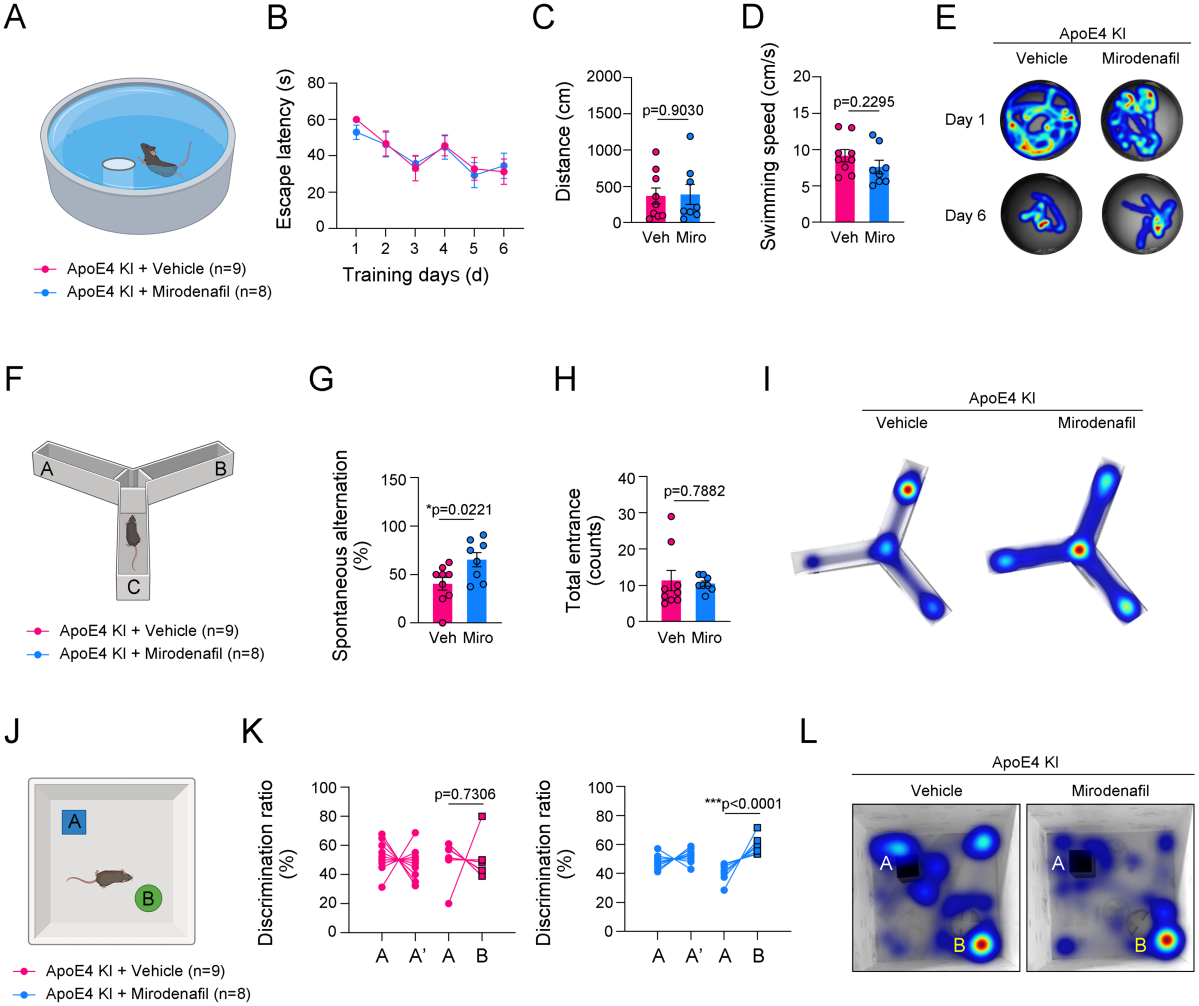
Mirodenafil improves the reduced short-term memory and novel object recognition in ApoE4 KI mice. **(A)** Illustration of Morris Water Maze (MWM). **(B–D)** The escape latencies, distance and swimming speed over 6 consecutive training days in ApoE4 KI + vehicle and ApoE4 KI + mirodenafil groups (*n* = 9 for ApoE4 KI + vehicle group and *n* = 8 for ApoE4 KI + mirodenafil groups). **(E)** Representative swimming paths between ApoE4 KI + vehicle, and ApoE4 KI + mirodenafil groups at 1st day and 6th day from the first training. **(F)** Illustration of Y-maze. **(G,H)** Percentage of spontaneous alteration and total counts of entrance in ApoE4 KI + vehicle and ApoE4 KI + mirodenafil groups (*n* = 9 for ApoE4 KI + vehicle group and *n* = 8 for ApoE4 KI + mirodenafil groups). **(I)** Representative movement paths between ApoE4 KI + vehicle, and ApoE4 KI + mirodenafil groups. **(J)** Illustration of Novel Object Recognition (NOR) test. **(K)** Discrimination ratio (%) before and after replacing with a novel object in ApoE4 KI + vehicle, and ApoE4 KI + mirodenafil groups (*n* = 9 for ApoE4 KI + vehicle group and *n* = 8 for ApoE4 KI + mirodenafil groups). **(L)** Representative movement paths between ApoE4 KI + vehicle, and ApoE4 KI + mirodenafil groups. Results are presented as mean ± SEM. Statistics were performed using two-sided Student’s *t*-test **(C,D,G,H,K)** and two-way ANOVA **(B)** followed by post hoc LSD test. *p < 0.05, **p < 0.01, and ***p < 0.001 between the indicated groups. The illustrations provided in panel **(A,F,J)** were created in BioRender.com.

We investigated whether cognitive and memory performance was impaired by ApoE4 KI and whether this could be regulated by mirodenafil administration. In the MWM test, mirodenafil-administered ApoE4 KI mice did not show a significant improvement in finding the platform compared to vehicle-administered mice (Figures 5A–E). However, in the Y-maze test, spontaneous alternation was significantly improved in mirodenafil-administered ApoE4 KI mice (Figures 5F–I). Moreover, in the NOR test, mirodenafil significantly improved the novel object recognition in ApoE4 KI mice (Figures 5J–L). These results indicated that cognitive function impaired by ApoE4 KI is improved by mirodenafil.

### 3.6 Mirodenafil enhanced endothelial cell stability and reduced classical activation of microglia

**Figure 6:**
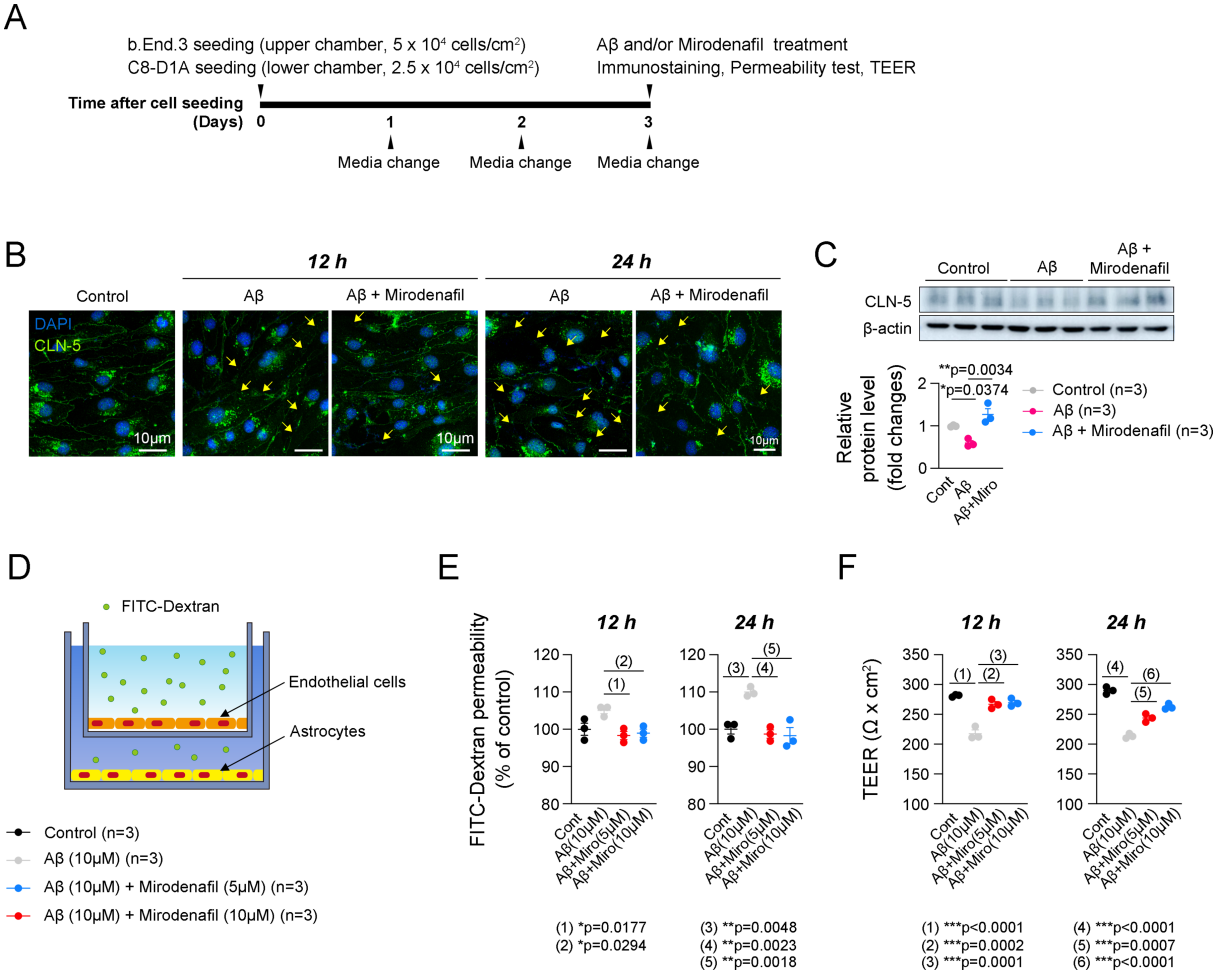
Mirodenafil reduces the endothelial cell permeability induced by Aβ_42_. **(A)** Experimental timetable for establishing an *in vitro* BBB model. **(B)** Representative images of CLN-5 staining in b.End.3 endothelial cells treated with or without Aβ_42_ or mirodenafil. Scale bars, 10 μm. **(C)** Western blotting data and quantification of CLN-5 in b.End.3 cells (*n* = 3). **(D)** A schematic diagram of the *in vitro* BBB model using b.End.3 endothelial cells and C8-D1A astrocyte cells. **(E)** Quantification of FITC-Dextran permeability in in b.End.3 endothelial cells treated with or without Aβ_42_ or mirodenafil (*n* = 3). **(F)** Quantification of TEER in b.End.3 endothelial cells treated with or without Aβ_42_ or mirodenafil (*n* = 3). Results are presented as mean ± SEM. Statistics were performed using two-sided Student’s *t*-test **(C-D, G-H, K)** and two-way ANOVA **(B)** followed by post hoc LSD test. *p < 0.05, **p < 0.01, and ***p < 0.001 between the indicated groups.

Lastly, we investigated the molecular mechanisms by which mirodenafil ameliorates histological damage. To determine how mirodenafil improves ApoE4-associated angiopathy, we conducted *in vitro* experiments using b.End.3 endothelial cells and C8-D1A astrocytes that replicate the BBB model (Figure 6A), as well as BV2 microglial cells. To verify whether mirodenafil directly exerts its beneficial effects through vascular stabilization, we treated Aβ_42_ with or without mirodenafil to b.End.3 cells, and then examined the changes in the expression of CLN-5. Immunostaining results showed decreased CLN-5 intensity and increased discontinuous CLN-5^+^ junctions after 12 and 24 h of Aβ_42_ treatment (Figure 6B). These phenomena were recovered by co-treatment with 10 μM mirodenafil (Figure 6B). Consistently, our western blotting results showed that the level of endothelial CLN-5 was reduced by Aβ_42_ treatment and restored by mirodenafil (Figure 6C). Since reduced CLN-5 expression was associated with increased cerebrovascular permeability and reduced blood perfusion (Argaw et al., 2009), we measured FITC-Dextran permeability in our *in vitro* BBB model (Figure 6D). As a result, endothelial cell permeability was increased by Aβ_42_ treatment and decreased by co-treatment with 5 μM and 10 μM mirodenafil (Figure 6E). Additionally, TEER, which reflects the integrity of endothelial cells, was decreased by Aβ_42_ treatment and recovered by mirodenafil co-treatment (Figure 6F). These results indicate that Aβ_42_ directly impairs BBB permeability and that these effects are significantly attenuated by mirodenafil.

**Figure 7:**
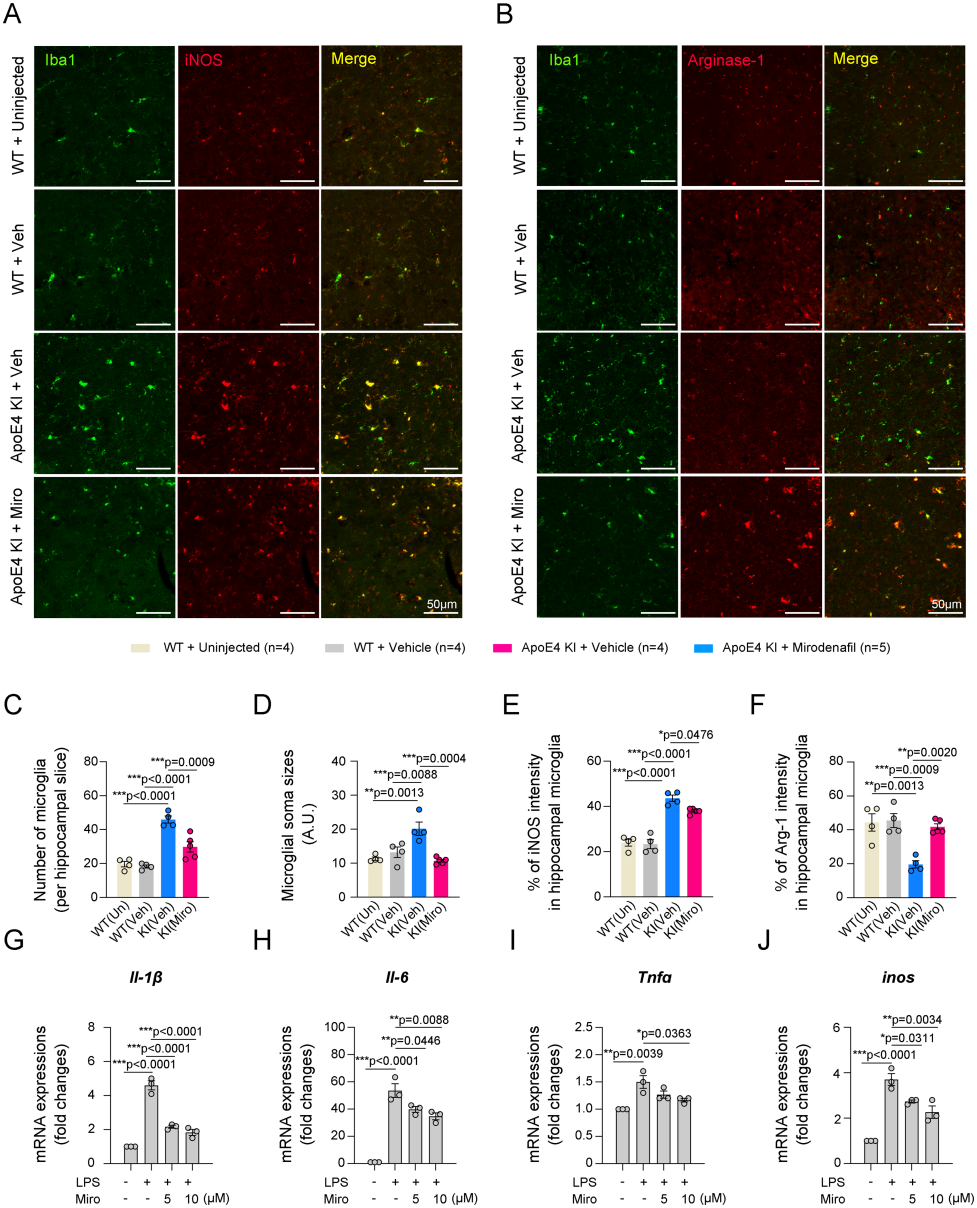
Mirodenafil reduces classical activation of microglia in the hippocampus of ApoE4 KI mice. **(A,B)** Representative images of double immunostaining for Iba1 and iNOS **(A)** or Iba1 and Arg-1 **(B)** in the hippocampus of uninjected WT, vehicle-administered WT and vehicle- or mirodenafil-administered ApoE4 KI mice. Scale bars, 50 μm. **(C–F)** Quantification of the number of microglia, intensities of microglial soma sizes, percentage of iNOS or Arg-1 in microglia in the hippocampus of vehicle-administered WT and vehicle- or mirodenafil-administered ApoE4 KI mice (*n* = 4 for uninjected WT, *n* = 4 for WT + vehicle and ApoE4 KI + vehicle groups, and *n* = 5 for ApoE4 KI + mirodenafil group). **(G–J)** Comparison of mRNA expressions of pro-inflammatory cytokines, including *Il-1β*, *Il-6*, *Tnfa*, and *inos* in BV2 microglial cells (*n* = 3). Results are presented as mean ± SEM. Statistics were performed using one-way ANOVA **(C–J)** followed by post hoc LSD test. *p < 0.05, **p < 0.01, and ***p < 0.001 between the indicated groups.

Classical activation of microglia in the hippocampus contributes to the progression of AD pathology through inflammatory responses, while alternative activation of microglia is known to have neuroprotective effects through anti-inflammatory responses (Wang et al., 2021; Guo et al., 2022). We observed that microglial activation in ApoE4 KI mice was significantly suppressed by mirodenafil administration to the level of uninjected or vehicle-administered WT mice (Figures 7A–D). Microglial iNOS expression indicates a pro-inflammatory classically activated state, while microglial Arg-1 expression indicates an anti-inflammatory alternatively activated state (Cherry et al., 2014). Our double-immunostaining data showed that the expression of iNOS was largely increased, while the expression of Arg-1 was reduced in the hippocampal microglia of ApoE4 KI mice (Figures 7A,B,E,F). Additionally, mirodenafil reduced microglial iNOS expression and increased Arg-1 expression in the hippocampus of ApoE4 KI mice (Figures 7A,B,E,F). Next, we investigated whether mirodenafil directly acts on microglia, thereby reducing classical activation of microglia. Thus, we activated BV2 microglial cells with lipopolysaccharide and examined whether mirodenafil co-treatment reduces the lipopolysaccharide-induced expression of pro-inflammatory cytokines, such as *Il-1β*, *Il-6*, *Tnfa*, and *Nos2*. Our real-time PCR data showed that lipopolysaccharide treatment significantly increased the expression of pro-inflammatory cytokines, which were dose-dependently reversed by co-treatment with mirodenafil (Figures 7G–J). These results indicate that mirodenafil induces anti-inflammation by reducing microglia’s classical activation.

## 4 Discussion

In the present study, we found that ApoE4 KI mice exhibited impairments in cerebrovascular perfusion, accumulation of Aβ_42_ plaques, and classical activation of microglia in the hippocampus. Additionally, ApoE4 KI mice showed deficits in short-term memory and cognitive function, as assessed by the Y-maze and NOR tests, respectively. Oral administration of mirodenafil for 4 weeks ameliorated the histopathological alterations induced by ApoE4 KI. The cognitive impairments observed in ApoE4 KI mice were significantly alleviated by mirodenafil administration. Lastly, in our *in vitro* experiments replicating the BBB, Aβ_42_ increased endothelial cell permeability and reduced CLN-5 expression, both of which were reversed by mirodenafil co-treatment. Furthermore, mirodenafil treatment significantly suppressed the expression of pro-inflammatory cytokines in lipopolysaccharide-treated BV2 microglial cells. Overall, these results suggest that mirodenafil can improve ApoE4-associated AD symptoms and may have therapeutic potential in patients with AD.

It has been known that approximately 40% of patients with AD have at least one copy of the ApoE4 allele (Corder et al., 1993; Safieh et al., 2019; Premkumar et al., 1996). Not only in AD, but ApoE4 is also associated with a high prevalence of cerebral amyloid angiopathy lesions at 32%, which is related to a reduction in CLN-5 expression (Premkumar et al., 1996). In our study, we observed A*β*_42_ accumulation, along with cerebral hypoperfusion, reduced CLN-5 expression, and decline in cognitive function in ApoE4 KI mice. These results indicated a strong association between Aβ_42_ accumulation and reduced cerebral blood flow. However, we failed to observe Aβ_42_ accumulation in the hypothalamus where vascular leakage frequently occurs in response to metabolic/inflammatory changes, such as fasting or a high-fat diet (Langlet et al., 2013; Lee et al., 2019). In addition, previous studies have shown that Aβ_42_ deposition mainly occurs in the hippocampus and cortex, rather than in the hypothalamus (Reilly et al., 2003; Hampel et al., 2021). Future studies are needed to investigate the mechanisms underlying cerebral hypoperfusion and Aβ_42_ accumulation in different brain regions.

Cerebral hypoperfusion is closely associated with AD progression. To address this, several approaches—such as using VEGF inhibitors to suppress vessel leakage or employing tPA to inhibit blood clotting—have been developed (Zhang et al., 2024; Uekawa et al., 2024). However, vascular-targeted research for AD treatment remains highly limited. Some studies have reported findings on repurposing PDE5i for AD treatment (Hainsworth et al., 2023); however, no study has yet demonstrated that PDE5i alleviates ApoE4-associated deficits. This study provides evidence that mirodenafil improves histological and behavioral alterations associated with the human ApoE4 allele. Our *in vitro* mechanistic study demonstrated that mirodenafil directly acts on endothelial cells and microglia, contributing to vascular stabilization and anti-inflammatory effects. In addition, we confirmed that mirodenafil administration in ApoE4 KI animals increased CREB phosphorylation in the hippocampus. CREB is crucial for cellular metabolism and survival and is particularly known for stabilizing and maintaining the endothelium (Huang et al., 2021; Watson et al., 2007). Moreover, in microglia, CREB phosphorylation is associated with anti-inflammatory effects by suppressing NF-κB signaling and enhancing the expression of anti-inflammatory cytokines such as interleukin-10 and transforming growth factor-beta (TGF-β) (Wen et al., 2010). The suppression of inflammation can also contribute to BBB stabilization. These findings align with previous studies showing that CREB signaling is reduced in neurodegenerative disease models such as AD and Parkinson’s disease and that increasing CREB phosphorylation can ameliorate these deficits (Pugazhenthi et al., 2011; Xu et al., 2022; Kim et al., 2020; Zhao et al., 2021). Since PDE5 inhibitors, such as sildenafil, vardenafil, and tadalafil are already FDA-approved drugs with partially validated safety profiles, they hold great potential as therapeutics.

We found that the Aβ_42_ accumulation was predominantly observed near the blood vessels of the hippocampus of ApoE4 KI mice indicating the possible association between the reduced cerebrovascular perfusion and AD progression (Mattsson et al., 2014). An intriguing observation was that the pattern of Aβ_42_ accumulation differs in the ApoE4 KI mouse model compared with other AD mouse models, such as APP/PS1 and 5xFAD mice (Locci et al., 2021). In APP/PS1 or 5xFAD mice, Aβ_42_ plaques are distributed throughout the parenchymal area of the cortex and hippocampus (Locci et al., 2021). In contrast, our study using ApoE4 KI mice revealed that plaques were seen around blood vessels and weekly in CA3 neurons. The differences in Aβ_42_ accumulation patterns across various AD models should be further investigated.

Inflammation in the central nervous system is closely associated with BBB dysfunction as well as AD progression (Obermeier et al., 2013; Zhang W. et al., 2023). In our study, ApoE4 KI mice induced microgliosis and increased the microglial iNOS expression, and decreased the microglial Arg-1 expression in the hippocampus. Moreover, mirodenafil reduced microglial iNOS expression and increased microglial Arg-1 expression indicating that mirodenafil reduced hippocampal inflammation induced by ApoE4 KI. These results are consistent with previous studies demonstrating the association between reduced microgliosis and improved cognitive function in patients with AD (Fan et al., 2015; Malpetti et al., 2020). Moreover, in our results using BV2 microglial cells, mirodenafil inhibited lipopolysaccharide-induced pro-inflammatory cytokines, such as *Il-1β*, *Il-6*, *Tnfα*, and *Nos2*. These results suggest that mirodenafil has a direct effect on microglia. Consistent with this, several studies have reported that sildenafil, another PDE5i, has direct anti-inflammatory effects (Zhao et al., 2011; Zych et al., 2019; Kniotek et al., 2021). In these studies, sildenafil treatment not only attenuates lipopolysaccharide-induced ROS-related mitogen-activated protein kinase (MAPK)/NF-κB signaling in the N9 microglial cell line (Zhao et al., 2011), but also reduced tumor necrosis factor-alpha (TNFα)-producing T cells and interferon-gamma (IFNγ) expression stimulated by phorbol myristate acetate in human peripheral blood mononuclear cells (Zych et al., 2019). These results suggest that PDE5 inhibitors, including mirodenafil, could be used in the future to alleviate inflammatory disorders.

Our behavioral tests found no significant difference in the MWM test between age-matched WT mice and ApoE4 KI mice. Cognitive impairment occurs depending on the mouse’s age or the types of AD models. Consistent with our findings, older ApoE4 KI mice (16 months old) also did not exhibit cognitive impairment as measured by the Morris Water Maze (MWM) (Leung et al., 2012). This suggests that the decline in spatial cognitive function occurs at more advanced stages of dementia compared to other indicators. Another factor is the possibility that different mechanisms may be involved, depending on genetic factors related to AD pathogenesis. The eNOS knockout mouse, another vascular dementia model, showed impaired recognition of a novel object; however, surprisingly, MWM performance was significantly improved (An et al., 2021; Frisch et al., 2000). Notable, both ApoE4 KI and eNOS KO models showed cerebral hypoperfusion (Tan et al., 2015). These results suggest that various cognitive impairments, vascular dysfunctions, and inflammatory disorders can occur in diverse AD models, highlighting the need for in-depth follow-up studies.

In conclusion, we demonstrated that mirodenafil can improve cognitive function by enhancing cerebrovascular perfusion, ameliorating classical activation of hippocampal microglia, and suppressing Aβ_42_ accumulation in ApoE4 KI mice. Furthermore, we suggest that mirodenafil has potential for future therapeutic applications in patients with AD.

## Data Availability

The datasets presented in this study can be found in online repositories. The names of the repository/repositories and accession number(s) can be found in the article/Supplementary material.

## Supplementary material

SUPPLEMENTARY FIGURE 1 Genotyping results for humanized ApoE4 KI mice.

SUPPLEMENTARY FIGURE 2 No difference in cerebrovascular perfusion in the hippocampus between male and female ApoE4 KI mice. Representative images quantification showing vessel intensities using FITC-Dextran in male and female ApoE4 KI mice (*n* = 4 for male and *n* = 3 for female). Scale bars, 200 μm. Results are presented as mean ± SEM. Statistics were performed using two-sided Student’s *t*-test. NS, not significant.

SUPPLEMENTARY FIGURE 3 Correlation between cerebrovascular perfusion and microgliosis in the hippocampus. Statistics were performed using simple linear regression.
