# Supplementary figures and images for: Mirodenafil improves cognitive function by reducing microglial activation and blood–brain barrier permeability in ApoE4 KI mice
Source: Front Aging Neurosci. 2025 May 15;17:1579411. doi: 10.3389/fnagi.2025.1579411 (PMC12119498; doi:10.3389/fnagi.2025.1579411)

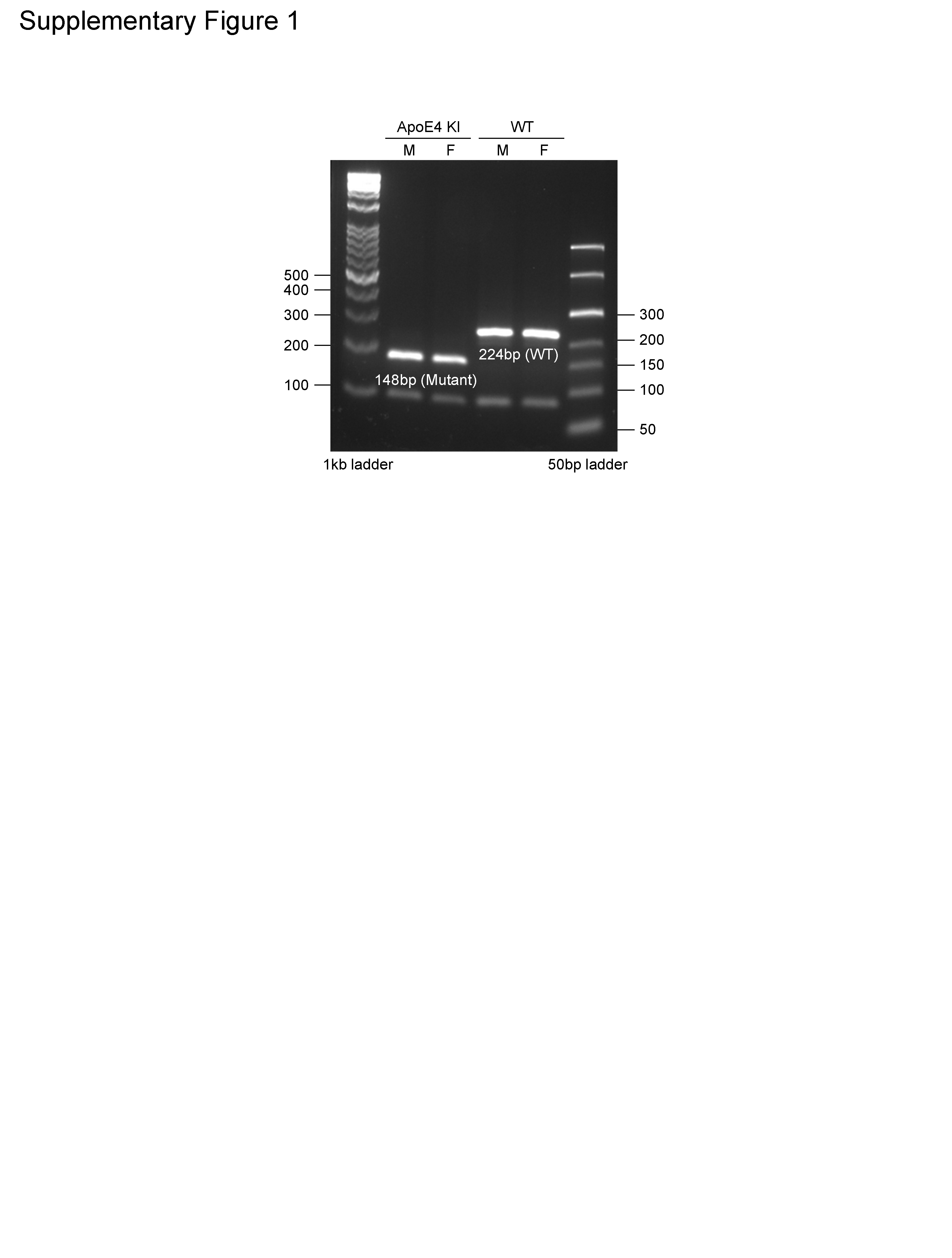

Supplement: SUPPLEMENTARY FIGURE 1 — Genotyping results for humanized ApoE4 KI mice. [file Image_1.tif]

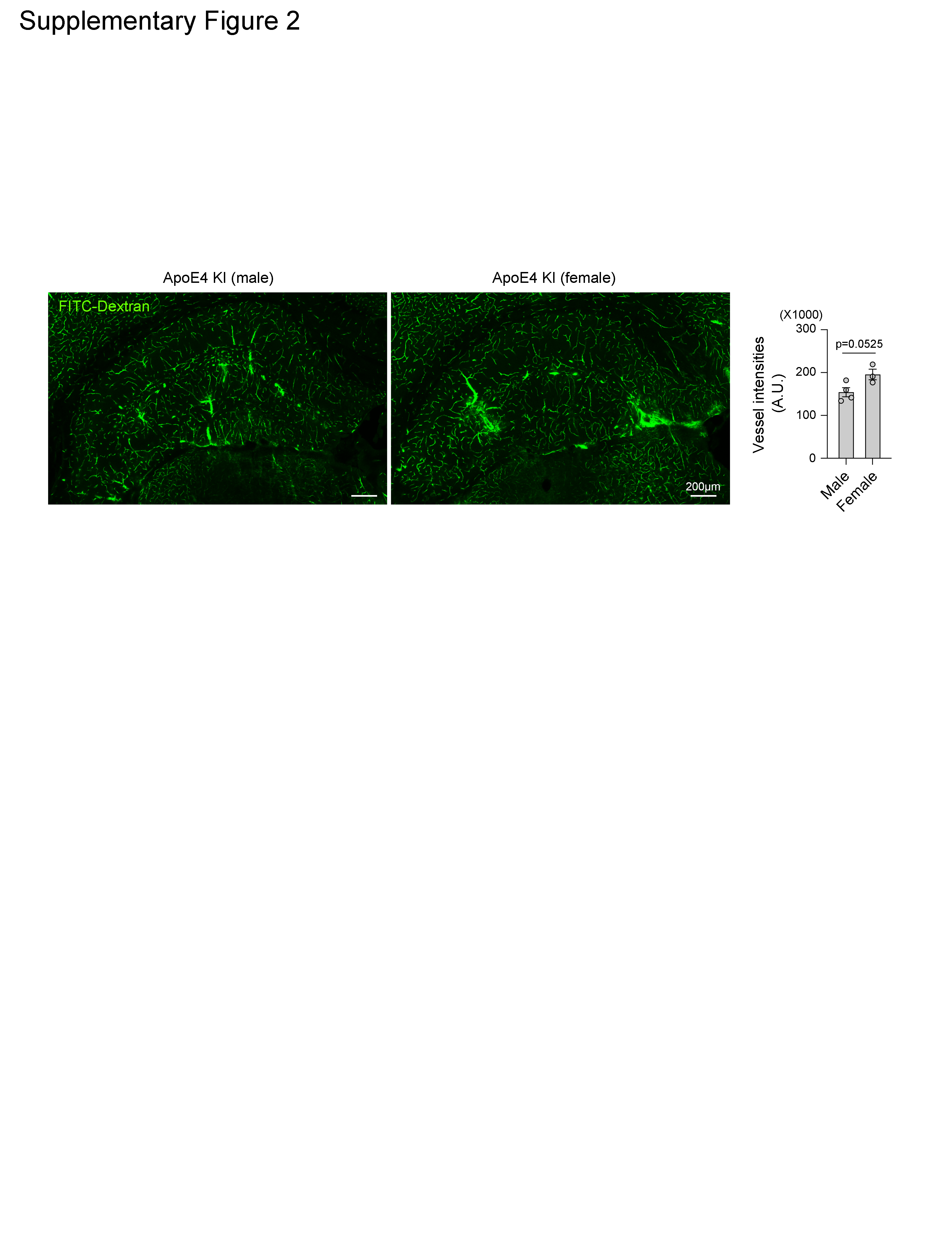

Supplement: SUPPLEMENTARY FIGURE 2 — No difference in cerebrovascular perfusion in the hippocampus between male and female ApoE4 KI mice. Representative images quantification showing vessel intensities using FITC-Dextran in male and female ApoE4 KI mice (n = 4 for male and n = 3 for female). Scale bars, 200 μm. Results are presented as mean ± SEM. Statistics were performed using two-sided Student’s t-test. NS, not significant. [file Image_2.tif]

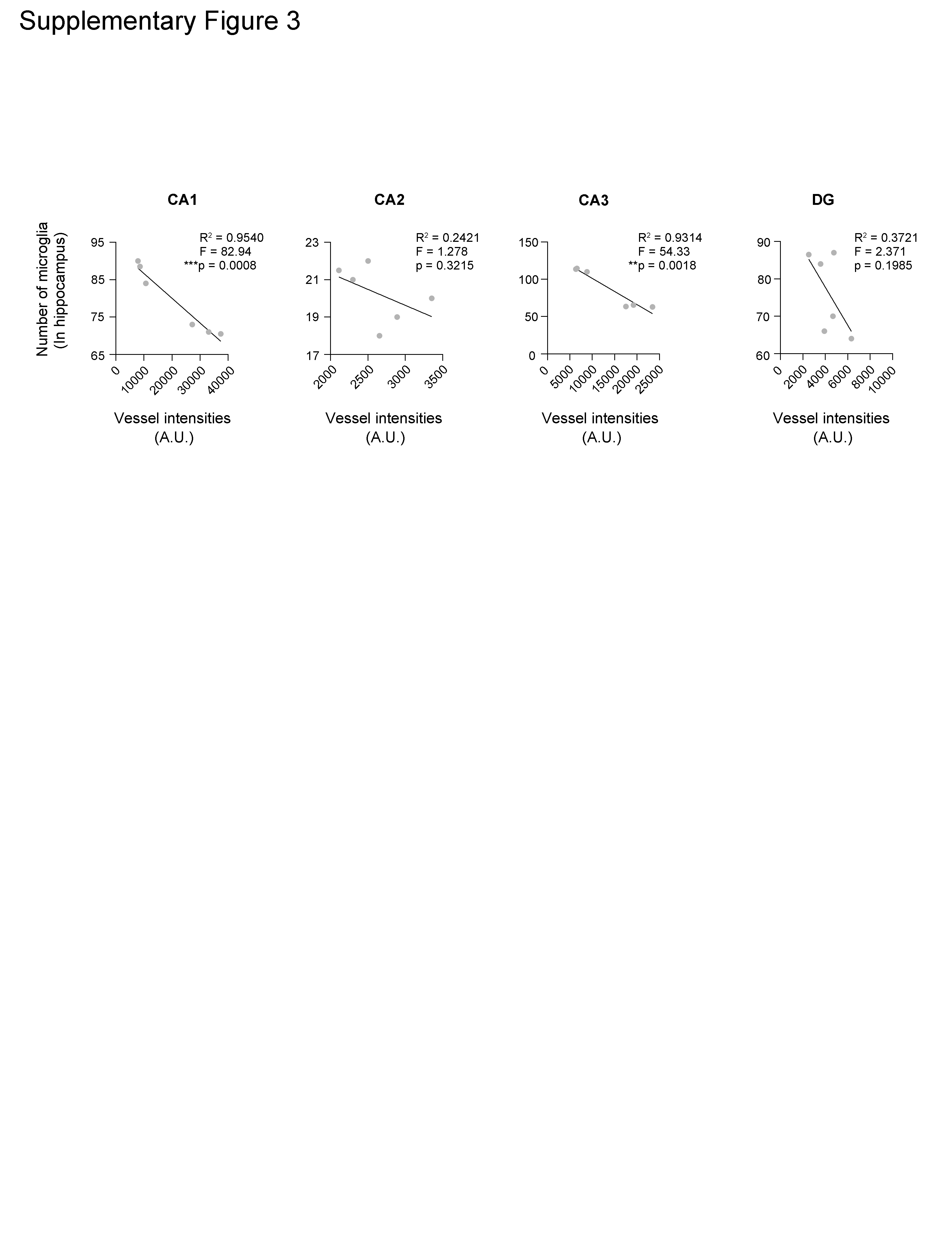

Supplement: SUPPLEMENTARY FIGURE 3 — Correlation between cerebrovascular perfusion and microgliosis in the hippocampus. Statistics were performed using simple linear regression. [file Image_3.tif]
